# Assessment of Follow-up Care After Emergency Department Presentation for Mild Traumatic Brain Injury and Concussion: Results From the TRACK-TBI Study

**DOI:** 10.1001/jamanetworkopen.2018.0210

**Published:** 2018-05-25

**Authors:** Seth A. Seabury, Étienne Gaudette, Dana P. Goldman, Amy J. Markowitz, Jordan Brooks, Michael A. McCrea, David O. Okonkwo, Geoffrey T. Manley, and the TRACK-TBI Investigators, Opeolu Adeoye, Neeraj Badjatia, Kim Boase, Yelena Bodien, M. Ross Bullock, Randall Chesnut, John D. Corrigan, Karen Crawford, Ramon Diaz-Arrastia, Sureyya Dikmen, Ann-Christine Duhaime, Richard Ellenbogen, V. Ramana Feeser, Adam Ferguson, Brandon Foreman, Raquel Gardner, Joseph Giacino, Luis Gonzalez, Shankar Gopinath, Rao Gullapalli, J. Claude Hemphill, Gillian Hotz, Sonia Jain, Frederick Korley, Joel Kramer, Natalie Kreitzer, Harvey Levin, Chris Lindsell, Joan Machamer, Christopher Madden, Alastair Martin, Thomas McAllister, Randall Merchant, Pratik Mukherjee, Lindsay Nelson, Florence Noel, Eva Palacios, Daniel Perl, Ava Puccio, Miri Rabinowitz, Claudia Robertson, Jonathan Rosand, Angelle Sander, Gabriella Satris, David Schnyer, Mark Sherer, Murray Stein, Sabrina Taylor, Nancy Temkin, Arthur Toga, Alex Valadka, Mary Vassar, Paul Vespa, Kevin Wang, John Yue, Esther Yuh, Ross Zafonte

**Affiliations:** 1 Department of Ophthalmology and Leonard D. Schaeffer Center for Health Policy and Economics, Keck School of Medicine, University of Southern California, Los Angeles; 2 Leonard D. Schaeffer Center for Health Policy and Economics, School of Pharmacy, University of Southern California, Los Angeles; 3 Leonard D. Schaeffer Center for Health Policy and Economics, Price School of Public Policy, University of Southern California, Los Angeles; 4 University of California, San Francisco; 5 Department of Neurological Surgery, University of Pittsburgh, Pittsburgh, Pennsylvania; 6 Department of Neurosurgery, Medical College of Wisconsin, Milwaukee; 7 Department of Neurological Surgery, University of California, San Francisco; 8 University of Cincinnati, Cincinnati, Ohio; 9 University of Maryland, Baltimore; 10 University of Washington, Seattle; 11 Massachusetts General Hospital, Boston; 12 University of Miami, Miami, Florida; 13 Ohio State University, Columbus; 14 University of Southern California, Los Angeles; 15 University of Pennsylvania, Philadelphia; 16 Massachusetts General Hospital for Children, Boston; 17 Virginia Commonwealth University, Richmond; 18 University of California, San Francisco; 19 Spaulding Rehabilitation Hospital, Charlestown, Massachusetts; 20 TIRR Memorial Hermann, Houston, Texas; 21 Baylor College of Medicine, Houston, Texas; 22 University of California, San Diego; 23 University of Michigan, Ann Arbor; 24 Vanderbilt University, Nashville, Tennessee; 25 University of Texas Southwestern Medical Center, Dallas; 26 Indiana University, Indianapolis; 27 Medical College of Wisconsin, Milwaukee; 28 Uniformed Services University, Bethesda, Maryland; 29 University of Pittsburgh, Pittsburgh, Pennsylvania; 30 University of Texas, Austin; 31 University of California, Los Angeles; 32 University of Florida, Gainesville; 33 Harvard Medical School, Boston, Massachusetts

## Abstract

**Importance:**

Mild traumatic brain injury (mTBI) affects millions of Americans each year. Lack of consistent clinical practice raises concern that many patients with mTBI may not receive adequate follow-up care.

**Objective:**

To characterize the provision of follow-up care to patients with mTBI during the first 3 months after injury.

**Design, Setting, and Participants:**

This cohort study used data on patients with mTBI enrolled in the Transforming Research and Clinical Knowledge in Traumatic Brain Injury (TRACK-TBI) study between February 26, 2014, and August 25, 2016. We examined site-specific variations in follow-up care, the types of clinicians seen by patients receiving follow-up care, and patient and injury characteristics associated with a higher likelihood of receiving follow-up care. The TRACK-TBI study is a prospective, multicenter, longitudinal observational study of patients with TBI presenting to the emergency department of 1 of 11 level I US trauma centers. Study data included patients with head trauma who underwent a computed tomography (CT) scan within 24 hours of injury, had a Glasgow Coma Scale score of 13 to 15, were aged 17 years or older, and completed follow-up care surveys at 2 weeks and 3 months after injury (N = 831).

**Main Outcomes and Measures:**

Follow-up care was defined as hospitals providing TBI educational material at discharge, hospitals calling patients to follow up, and patients seeing a physician or other medical practitioner within 3 months after the injury. Unfavorable outcomes were assessed with the Rivermead Post Concussion Symptoms Questionnaire.

**Results:**

Of 831 patients (289 [35%] female; 483 [58%] non-Hispanic white; mean [SD] age, 40.3 [16.9] years), less than half self-reported receiving TBI educational material at discharge (353 patients [42%]) or seeing a physician or other health care practitioner within 3 months after injury (367 patients [44%]). Follow-up care varied by study site; adjusting for patient characteristics, the provision of educational material varied from 19% to 72% across sites. Of 236 patients with a positive finding on a CT scan, 92 (39%) had not seen a medical practitioner 3 months after the injury. Adjusting for injury severity and demographics, patient admission to the hospital ward or intensive care unit, patient income, and insurance status were not associated with the probability of seeing a medical practitioner. Among the patients with 3 or more moderate to severe postconcussive symptoms, only 145 of 279 (52%) reported having seen a medical practitioner by 3 months.

**Conclusions and Relevance:**

There are gaps in follow-up care for patients with mTBI after hospital discharge, even those with a positive finding on CT or who continue to experience postconcussive symptoms.

## Key Points

**Question:**

Do patients with mild traumatic brain injury (mTBI) receive adequate levels of follow-up care?

**Findings:**

In a cohort study using data on 831 patients with mTBI presenting to the emergency department at 1 of 11 level I trauma centers across the United States, 42% of patients reported receiving educational material at discharge and 44% reported seeing a physician or other medical practitioner within 3 months after injury. Among patients with 3 or more moderate to severe postconcussive symptoms, only 52% reported having seen a practitioner within 3 months following the injury.

**Meaning:**

A large proportion of patients with mTBI do not receive follow-up care after injury even when they experience ongoing postconcussive symptoms.

## Teaser

This cohort study describes follow-up care for patients within 3 months of receiving treatment for mild traumatic brain injury (TBI) or concussion at US emergency departments of level I trauma centers.

## Introduction

Traumatic brain injury (TBI) is a critical global public health issue. This type of injury affects millions of Americans each year, resulting in approximately 2.5 million emergency department (ED) visits in 2013.^1^ Of brain injuries among patients presenting to the ED, most are classified as mild TBI (mTBI) or concussion, defined by an initial Glasgow Coma Scale (GCS) score of 13 to 15.^2,3^ Despite the classification of *mild,* mTBI can lead to persistent physical, neuropsychiatric, and cognitive symptoms that have a major impact on function and quality of life of the injured patient.^4,5,6,7^ In a recent report from the Transforming Research and Clinical Knowledge in Traumatic Brain Injury (TRACK-TBI) study, 22% of patients with mTBI remained functionally impaired 1 year after the injury.^4^ While data on the cost of mTBI are limited, one estimate suggests that mTBI is associated with as much as a 75% increase in expected medical costs up to 3 years after injury,^8^ and there is growing evidence that the sequelae of TBI may also contribute to loss of employment, homelessness, and incarceration.^9,10,11^

While patients with moderate to severe TBI are almost always admitted to a hospital or intensive care unit (ICU) for close monitoring and intervention,^12,13,14^ there is considerably less consensus as to best practices for patients with mTBI.^15,16,17,18^ The lack of consistent clinical practice raises concerns that many patients with mTBI may not receive adequate follow-up care. To date, few studies have investigated follow-up care after mTBI, but what evidence does exist suggests important deficiencies. For instance, more than 60% of a sample of patients that included both those with mTBI and those with moderate to severe TBI (GCS score of 3-15) received no additional services following discharge from the acute care hospital in the late 1990s.^19^ Failure to follow up with patients could have adverse consequences, as simply providing educational materials to patients with mTBI is associated with improved outcomes.^20,21,22^

In the current study, we examined a key aspect of mTBI and concussion management, the provision of follow-up care after hospital discharge, using data from the prospective, multicenter, longitudinal observational TRACK-TBI study cohort. Participants in this study were surveyed regarding follow-up care in the first 3 months after injury. We examined site-specific variations in follow-up care, the types of physicians or other medical practitioners seen, and the patient and injury characteristics associated with a higher likelihood of receiving follow-up care.

## Methods

### Study Data

The TRACK-TBI study is an ongoing prospective, longitudinal, observational study of patients with TBI who presented to the ED of 1 of 11 level I US trauma centers (Ben Taub General Hospital, Houston, Texas; Massachusetts General Hospital, Boston; Zuckerberg San Francisco General Hospital, San Francisco, California; University of Cincinnati Medical Center, Cincinnati, Ohio; R Adams Cowley Shock Trauma Center, Baltimore, Maryland; Ryder Trauma Center, Miami, Florida; University of Pittsburgh Medical Center, Pittsburgh, Pennsylvania; Seton Medical Center, Austin, Texas; Parkland Memorial Hospital, Dallas, Texas; Harborview Medical Center, Seattle, Washington; and Virginia Commonwealth University Medical Center, Richmond).

Participants or their legal representatives provided written informed consent to participate. Competency for self-consent was determined through administration of the Galveston Orientation and Amnesia Test. For those without a passing score, a legally authorized representative provided consent, and competency screening was completed at subsequent follow-ups. Eligible participants were approached by study staff who explained the study, reviewed the consent form, and obtained consent within 24 hours of injury, unless the site had a waiver of consent for enrollment. A number of TRACK-TBI sites obtained local institutional review board approval to collect biospecimens along with a defined set of acute data variables under a waiver of consent. A waiver of consent was applied in situations in which a potential study patient satisfied all study inclusion criteria but did not have the capacity to consent and did not have a legally authorized representative who could be identified within the 24-hour consent period required by the study. This type of circumstance occurs with some frequency in this population because of the rapidly unfolding events around these traumatic injuries. This waiver of consent protocol allowed collection of vitally important biospecimen data during the acute time frame, a period when the brain and body are experiencing rapidly fluctuating reactions due to the injury. Informed consent was obtained when it was determined that the patient regained capacity to consent or a legally authorized representative was identified. In a small number of special cases, the local institutional review board approved biospecimen data retention in the study without informed consent from the patient or a legally authorized representative (eg, patient died and a legally authorized representative was never identified due to homelessness, no family, etc). All data analyzed in this study were stripped of protected health information and completely deidentified. The institutional review boards of each institution approved the study. The Strengthening the Reporting of Observational Studies in Epidemiology (STROBE) reporting guideline was followed.

Inclusion criteria were acute head trauma sufficient for an ED physician to order a clinical head computed tomography (CT) scan within 24 hours of injury. Exclusion criteria included pregnancy, incarceration, nonsurvivable physical trauma, debilitating mental health disorders or neurological disease, magnetic resonance imaging contraindications (eg, cardiac pacemakers, aneurism clips, insulin pumps), and preexisting medical conditions that could interfere with outcome assessments. Because this study focused on mTBI, including concussion, we further restricted the sample to patients with a GCS score of 13 to 15 on arrival at the ED, loss of consciousness for less than 30 minutes, and posttraumatic amnesia duration of less than 24 hours. At each milestone (2 weeks and 3 months), participants were followed up with 19 standard outcome assessments that included measures of postconcussive and/or TBI-related symptoms, psychological health, and global outcome. In addition, a 200-question interview was administered to participants or their surrogates to assess follow-up medical care as well as the economic and health consequences of TBI.

Study data analyzed in this article include TRACK-TBI patients aged 17 years or older with mTBI who were enrolled between February 26, 2014, and August 25, 2016. Patient characteristics used in the analyses included age, sex, race and ethnicity, health insurance status, and income prior to injury. These variables were used as covariates in statistical analyses to account for any observable differences in the patient population that might correlate with follow-up care. The data also included injury characteristics, including admission GCS score, initial head CT scan results, cause of injury, and whether the patient experienced posttraumatic amnesia, loss of consciousness, or altered consciousness. Patients were identified as having a positive finding on the initial head CT if a lesion consistent with acute TBI (eg, contusion, subarachnoid hemorrhage) was documented in the radiology report.

The primary goal of the analysis was to examine patients’ receipt of care and how it varied according to patient characteristics and across study sites. The 2-week and 3-month follow-up surveys captured self-reports of follow-up care received and clinical outcome measures of physical and psychological symptom burden. Specifically, patients were asked whether they received TBI educational materials from the hospital at discharge, whether anyone from the hospital called them to follow up about their TBI, and whether they had seen a physician or other health care practitioner since being discharged (and if so, the type of practitioner seen). Our analysis only included patients who responded to all interview questions pertaining to follow-up care at both 2 weeks and 3 months.

To measure clinical outcomes, patients in the TRACK-TBI study were assessed with the Rivermead Post Concussion Questionnaire (RPQ) and the Brief Symptom Inventory-18 (BSI-18) tool at 3 months. The RPQ measures severity of headaches, dizziness, and nausea as well as cognitive, mood, and sleep disturbances and other physical symptoms associated with postconcussion syndrome (PCS) on a scale of 0 to 4, with 0 indicating the symptom was not experienced at all and 4 indicating the symptom was a severe problem within the past 7 days, as compared with preinjury status. Following Sterr et al,^23^ we identified patients with PCS as those with 3 or more postconcussive symptoms with a severity rating of 3 or higher. The BSI-18 provides an assessment of psychological distress by measuring depression, anxiety, and somatization symptom domains. This study used *t* scores, calculated from the summed responses in each domain and normalized across sex. A Global Severity Index *t* score of 63 or higher is considered a clinically significant level of distress.^4^

### Statistical Analysis

We first conducted a descriptive analysis by measuring the percentage of patients who reported receiving educational materials at discharge, receiving a follow-up call from the hospital, or seeing a medical practitioner in the 2 weeks after injury and in the 3 months after injury. We compared follow-up care for patients with and without positive findings on the initial head CT to test whether the likelihood of follow-up care was associated with clinical severity. For those patients who did see a practitioner by 3 months, we examined the type(s) of practitioners seen.

Differences in the rate of follow-up care across study sites could be driven by differences in the underlying patient characteristics (eg, patient insurance status). To account for this, we used multivariate logistic regression to test whether patient characteristics, injury characteristics, or study site were associated with the receipt of follow-up care. We examined odds ratios (ORs) from the logistic regression to examine how patient and injury characteristics were associated with follow-up care. To demonstrate the magnitude of variation in follow-up care across study sites, we computed adjusted values for the percentage of patients expected to receive follow-up care in each site holding other covariates at their mean values. The variation in these adjusted values reports the site-specific variation in the use of follow-up care, removing any observable differences in the underlying patient populations across sites.

Finally, we investigated whether receipt of follow-up care was correlated with outcomes at 3 months. We tested whether patients who were symptomatic and experiencing worse outcomes were more likely to receive follow-up care by calculating the percentage of patients who reported having seen a medical practitioner by 3 months after injury compared with their burden of postconcussive symptoms and psychological distress. The significance of the differences in the percentage of patients seeing a medical practitioner by 3 months for those who were or were not symptomatic was assessed using paired *t* tests.

As noted previously, we excluded patients who either exited the study or did not respond to the questions about educational materials or seeing a medical practitioner at either 2 weeks or 3 months. This exclusion could have been because patients were lost to follow-up, because the full battery was not completed, or because the follow-up care variables did not apply (for example, if participants were still admitted at 2 weeks, the follow-up care questions were not administered). To understand whether this exclusion resulted in selection bias in the study sample, we compared patient and injury characteristics of the full sample and the final study sample. We conducted a multivariate logistic regression of the likelihood of being included in the study to determine whether demographic, socioeconomic, or injury factors were associated with missingness.

Stata statistical software version 14.0 (StataCorp) was used for all statistical analyses.

## Results

There were 1316 patients with mTBI enrolled in the TRACK-TBI study at the point that we began our analysis. Of these, 1017 (77%) completed all follow-up care questions at 2 weeks and 919 (70%) completed them at 3 months; we further restricted the sample to participants who had completed follow-up care questions at both time points, resulting in a final study sample of 831 participants (289 [35%] female; 483 [58%] non-Hispanic white; mean [SD] age, 40.3 [16.9] years). Most patients (460 [55%]) had an annual income of less than $50 000. The eTable in the Supplement compares characteristics of the full sample and the study sample. Overall, patient characteristics were similar between the full sample and the study sample. Using multivariate logistic regression to test which variables were associated with inclusion in the final study sample, we found that privately insured patients were more likely to be included than uninsured patients (OR, 1.81; 95% CI, 0.39-0.79), while patients with an undisclosed or unknown income level were less likely to be included than patients with a reported income of less than $50 000 (OR, 0.62; 95% CI, 0.45-0.84). Admission to the ICU was also associated with a lower likelihood of being included (OR relative to patients discharged from the ED, 0.47; 95% CI, 0.32-0.69), at least in part because these patients were still being cared for in the acute setting and were therefore less likely to be administered the questions at 2 weeks.

We describe the characteristics of the final study sample in Table 1. Of the 831 patients in the sample, 658 (79%) had a GCS score of 15, corresponding to the highest level of consciousness indicator. Overall, 236 patients (28%) had a lesion on the initial CT scan. Approximately 35% (288 patients) were discharged home, 44% (362 patients) were admitted to the hospital but not to the ICU, and 22% (181 patients) were admitted to the ICU.

**Table 1.** Summary Statistics

Patient Characteristic	All Patients Included in the Study (N = 831)^a^	Patients With a Positive Finding on CT Scan (n = 236)	Patients With ≥3 Moderate or Severe Postconcussion Symptoms (n = 279)^b^
Patient demographic characteristics			
Female, No. (%)	289 (35)	70 (30)	127 (46)
Non-Hispanic white, No. (%)	483 (58)	144 (61)	137 (49)
Age, mean (SD), y	40.3 (16.9)	44 (18.2)	40.6 (14.9)
Age 17-64 y, No. (%)	743 (89)	195 (83)	262 (94)
Age ≥65 y, No. (%)	88 (11)	41 (17)	17 (6)
Income and insurance			
Annual income <$50 000, No. (%)	460 (55)	129 (55)	158 (57)
Annual income ≥$50 000, No. (%)	212 (26)	72 (31)	54 (19)
Unknown income, No. (%)	159 (19)	35 (15)	67 (24)
Uninsured (self-pay), No. (%)	153 (18)	46 (20)	58 (21)
Insured, No. (%)	678 (82)	190 (81)	221 (79)
Private insurance, No. (%)	468 (56)	125 (53)	146 (52)
Medicaid, No. (%)	85 (10)	14 (6)	41 (15)
Medicare, No. (%)	61 (7)	28 (12)	15 (5)
Injury severity			
Lesion detected on CT scan, No. (%)	236 (28)	236 (100)	64 (23)
GCS score at arrival, mean (SD)	14.8 (0.5)	14.7 (0.5)	14.8 (0.5)
Patients with GCS score of 13, No. (%)	25 (3)	6 (3)	12 (4)
Patients with GCS score of 14, No. (%)	148 (18)	53 (23)	43 (15)
Patients with GCS score of 15, No. (%)	658 (79)	177 (75)	224 (80)
Mechanism of injury			
Road traffic incident, No. (%)	494 (59)	120 (51)	172 (62)
Incidental fall, No. (%)	203 (24)	79 (34)	53 (19)
Violence or assault, No. (%)	52 (6)	20 (9)	25 (9)
Care disposition			
ED discharge, No. (%)	288 (35)	21 (9)	97 (35)
Hospital admission, no ICU, No. (%)	362 (44)	106 (45)	120 (43)
Hospital admission, ICU, No. (%)	181 (22)	109 (46)	62 (22)

Abbreviations: CT, computed tomography; ED, emergency department; GCS, Glasgow Coma Scale; ICU, intensive care unit.

^a^ Patients were excluded from the study if they did not complete follow-up surveys 2 weeks and 3 months after injury.

^b^ Postconcussive symptoms were measured using the Rivermead Post Concussion Symptoms Questionnaire 3 months after injury.

Most patients with mTBI and concussion enrolled in the TRACK-TBI study received no follow-up care at either 2 weeks or 3 months after injury (Table 2). Approximately 42% (353 patients) reported that they received TBI educational material at discharge and 27% (209 patients) reported having been called to follow up about their brain injury by 2 weeks. Similarly, less than half of patients (41% [343 patients]) reported having seen a medical practitioner about their mTBI at 2 weeks, and 44% (367 patients) reported seeing a medical practitioner by 3 months.

**Table 2.** Proportion of Patients Reporting Follow-up Care After Injury^a^

Type of Follow-up Care	All Patients (N = 831)	Patients With Lesion Detected or Suspected on CT Scan (n = 236)	Patients With No Lesion Detected or Suspected on CT Scan (n = 595)
Received TBI educational material at discharge, No. (%)^b^	353 (42)	110 (49)	243 (46)
Hospital called to follow up by 2 wk, No. (%)^b^	209 (27)	55 (24)	154 (28)
Saw a practitioner by 2 wk, No. (%)	343 (41)	132 (56)	211 (35)
Saw practitioner by 3 mo, No. (%)	367 (44)	144 (61)	223 (37)

Abbreviations: CT, computed tomography; TBI, traumatic brain injury.

^a^ Unadjusted means.

^b^ Proportions of patients who received TBI educational material at discharge and whom the hospital called to follow up by 2 weeks exclude patients who answered “unknown” to these questions (n = 82 and n = 43, respectively).

There was wide site-specific variation in patients’ receipt of follow-up care even after adjusting for patient characteristics across sites. The Figure depicts the adjusted rates of TBI educational material receipt at discharge and having seen a medical practitioner by 3 months. These rates correspond to the expected percentage of patients receiving follow-up care at each site if all patients had identical demographics and injury characteristics. Sites were ranked by the adjusted rate of educational material receipt and assigned an alphabetic identifier for display purposes (A-K). Two sites, J and K, differed from the others in that they had affiliated specialty TBI clinics. The adjusted rate of receiving educational material at discharge varied from 19% to 72%, while the rate of having seen a practitioner at 3 months after injury ranged from 22% to 58%. There was no discernable site-level correlation between receiving educational materials and seeing a practitioner, except that sites with specialty TBI clinics had the highest rates for both outcomes.

**Figure.**
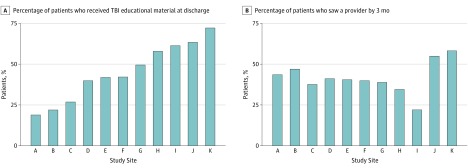
Adjusted Outcomes for Patients With Mild Traumatic Brain Injury (TBI) There was wide site-specific variation in patients’ receipt of follow-up care even after adjusting for patient characteristics across sites. The graphs show the adjusted rates of TBI educational material receipt at discharge (A) and having seen a medical practitioner by 3 months (B). The adjusted rate of receiving educational material at discharge varied from 19% to 72%, while the rate of having seen a practitioner at 3 months after injury ranged from 22% to 58%.

Of the 273 patients who saw a medical practitioner by 3 months and answered the series of follow-up questions to identify the type of practitioner, 218 (80%) reported that seeing a clinician was helpful. By 3 months, slightly less than a third (29% [80 patients]) had seen more than 1 clinician type. The majority (52% [141 patients]) reported seeing a general practitioner, while 38% (105) saw a neurologist (Table 3). Of particular interest to our analysis was the percentage of patients who visited a TBI clinic, where they would have been most likely to see a practitioner specializing in TBI care. Only 15% (42 patients) reported visiting a clinic that specialized in TBI or concussion. Of the 42 patients who reported having visited a TBI clinic, 31 (74%) were treated at sites J or K, although these study sites represent just 24% of the full study sample.

**Table 3.** Type of Clinician Care Seen by 3 Months After Injury^a^

Clinician Type	%
General practitioner	52
Neurologist	38
TBI or concussion clinic	15
Psychologist	5
Alternative medicine practitioner	3
Chiropractor	3
Physiatrist	2
Psychiatrist	2
Another type of practitioner	22

Abbreviation: TBI, traumatic brain injury.

^a^ Unadjusted percentages among patients who saw a practitioner by 3 months after injury. Patients could identify more than 1 type of clinician. Ninety-four patients who said they saw a practitioner but did not identify the practitioner type were classified as having seen an unknown practitioner type and were excluded from these percentages.

Injury severity was associated with patient receipt of follow-up care (Table 4). In particular, a GCS score of 13 or 14, denoting a higher injury severity than a score of 15 (OR, 1.69; 95% CI, 1.10-2.58) and a positive finding on the initial head CT scan consistent with TBI (OR, 2.95; 95% CI, 1.90-4.60) were associated with greater likelihood of seeing a medical practitioner. By 3 months, 144 of the 236 patients with a positive finding on a CT scan (61%) had seen a medical practitioner and 92 (39%) had not. In contrast, loss of consciousness and alteration of consciousness were not associated with having seen a medical practitioner, nor were hospital admission or admission to the ICU.

**Table 4.** Odds Ratios of Reporting Having Seen a Practitioner by 3 Months After Injury by Patient and Injury Characteristics

Patient and Injury Characteristics	No. With Event/Total No. (%)	Adjusted Odds Ratio (95% CI)^a^
Patient demographics		
Age 17-64 y	322/743 (43.3)	1 [Reference]
Age ≥65 y	45/88 (51.1)	1.22 (0.57-2.62)
Male	228/542 (42.1)	1 [Reference]
Female	139/289 (48.1)	1.44 (1.01-2.08)
Other racial/ethnic group	122/348 (35.1)	1 [Reference]
Non-Hispanic white	245/483 (50.7)	1.64 (1.10-2.43)
Income and insurance		
Annual income <$50 000	193/460 (42.0)	1 [Reference]
Annual income ≥$50 000	112/212 (52.8)	1.35 (0.88-2.07)
Annual income unknown	62/159 (39.0)	0.87 (0.55-1.37)
Uninsured (self-pay)	51/153 (33.3)	1 [Reference]
Private insurance	223/468 (47.6)	1.64 (0.98-2.74)
Medicaid	34/85 (40.0)	1.46 (0.73-2.92)
Medicare	28/61 (45.9)	0.90 (0.33-2.41)
Other or unknown insurance type	31/64 (48.4)	1.48 (0.72-3.04)
Injury severity		
No lesion suspected on CT scan	223/595 (37.5)	1 [Reference]
Lesion detected or suspected on CT	144/236 (61.0)	2.95 (1.90-4.60)
GCS score of 15	273/658 (41.5)	1 [Reference]
GCS score of 13 or 14	94/173 (54.3)	1.69 (1.10-2.58)
No posttraumatic amnesia	65/168 (38.7)	1 [Reference]
Posttraumatic amnesia	267/583 (45.8)	0.95 (0.61-1.47)
No loss of consciousness	50/120 (41.7)	1 [Reference]
Loss of consciousness	299/673 (44.4)	1.07 (0.66-1.76)
No alteration of consciousness	61/148 (41.2)	1 [Reference]
Alteration of consciousness	266/585 (45.5)	1.27 (0.82-1.97)
Mechanism of injury		
Road traffic incident	203/494 (41.1)	1 [Reference]
Incidental fall	102/203 (50.2)	1.05 (0.69-1.61)
Violence or assault	23/52 (44.2)	1.68 (0.82-3.41)
Other	39/82 (47.6)	1.35 (0.76-2.39)
Care disposition		
ED discharge	105/288 (36.5)	1 [Reference]
Hospital admission, no ICU	167/362 (46.1)	1.08 (0.70-1.67)
Hospital admission, ICU	95/181 (52.5)	1.05 (0.60-1.86)
Study site		
A	34/76 (44.7)	1 [Reference]
B	36/82 (43.9)	1.15 (0.35-3.78)
C	22/48 (45.8)	0.78 (0.30-2.01)
D	23/71 (32.4)	0.91 (0.40-2.04)
E	57/112 (50.9)	0.88 (0.43-1.82)
F	31/86 (36.0)	0.86 (0.37-2.00)
G	9/28 (32.1)	0.82 (0.29-2.29)
H	34/89 (38.2)	0.68 (0.29-1.59)
I	11/39 (28.2)	0.37 (0.13-1.04)
J	56/95 (58.9)	1.58 (0.72-3.49)
K	54/105 (51.4)	1.81 (0.87-3.75)

Abbreviations: CT, computed tomography; ED, emergency department; GCS, Glasgow Coma Scale; ICU, intensive care unit.

^a^ Adjusted odds ratios and confidence intervals calculated after the estimation of a multivariate logistic model.

Some patient demographic characteristics were associated with follow-up care. Women (OR, 1.44; 95% CI, 1.01-2.08) and non-Hispanic white patients (OR, 1.64; 95% CI, 1.10-2.43) were more likely than men and members of other racial/ethnic groups, respectively, to have seen a practitioner by 3 months. However, when controlling for these factors, age, patient admission to the hospital ward or ICU, income, and insurance status were not associated with having seen a medical practitioner by 3 months.

Patients with poorer outcomes 3 months after injury were only somewhat more likely to receive follow-up care. At the 3-month follow-up, 279 patients (34%) had 3 or more moderate or severe postconcussive symptoms on the RPQ, consistent with PCS.^23^ Among the patients with PCS, 145 (52%) reported having seen a practitioner by 3 months, with the remainder (134 patients [48%]) reporting no follow-up. Similarly, for the 145 patients (17%) with BSI-18 scores meeting definitions of psychological distress, less than half (68 patients [47%]) had seen a practitioner by 3 months.

## Discussion

We found that less than half of patients with mTBI self-reported receiving TBI educational material at discharge or seeing a medical practitioner within 3 months of the injury. There were wide disparities in rates of follow-up care across study sites and variation among the types of medical practitioners who provide follow-up care to patients with mTBI. Overall, a positive finding on the initial head CT scan was most closely associated with receiving follow-up care. Nevertheless, nearly 40% of patients with a positive finding on a head CT scan (92 of 236) had not seen a physician or other practitioner by 3 months following their injury. Somewhat surprisingly, patient income and insurance status were not associated with receipt of follow-up care. We also found that even patients admitted to the hospital ward or ICU were no more likely to have received follow-up care than those discharged directly from the ED. The finding that 48% of patients with significant postconcussive symptoms (134 of 279) were not seen by a medical practitioner by 3 months underscores the need to improve the system of care for mTBI and concussion.

Low rates of follow-up care, in and of themselves, do not necessarily signify an unmet need for care. If patients who did not see a practitioner were those who fully recovered from their TBI, the observed rates of care might be reasonable. However, our findings suggest that this is probably not the case. Despite higher rates of follow-up care for patients with a lesion on a CT scan, only half (145 of 279 [52%]) of patients with 3 or more moderate to severe postconcussive symptoms on the RPQ had seen a practitioner by 3 months after their injury.

Our findings reveal the consequences that may result from the absence of systems of follow-up care for patients with mTBI and concussion. They also highlight an apparent lack of appreciation by many clinicians of the substantial symptom and life burdens experienced by a significant proportion of patients with injuries labeled mild. One contributing factor in the acute and critical care setting is that mTBI comes with extremely low risk of mortality. For that reason, patients with mTBI are often quickly triaged. Increased efforts are warranted to raise ED clinician awareness of the importance of follow-up care to prevent morbidity and disability.

Recent evidence has stressed that mild TBI can have serious consequences and is often associated with burdensome symptoms and disabilities.^4,24,25,26^ Indeed, for a third of the patients included in this study (279 patients [34%]), 3 or more moderate to severe postconcussive symptoms persisted 3 months after injury. Such outcomes emphasize the need for better—and more consistent—care for a population at high risk of long-term disability and quality-of-life impairments. It is noteworthy that our results, reported in the Figure, show that patients treated at the 2 facilities with specialty TBI clinics fared the best as measured by receiving educational materials and seeing a practitioner by 3 months.

Although we are not aware of any other recent studies examining rates of follow-up care for patients with mTBI at level I trauma centers across the United States, our study can be grouped with related literature examining the value of postacute care. Driven in part by new programs imposing penalties for hospital readmission, there has been considerable interest in testing whether follow-up after discharge can prevent readmission, although results have been mixed.^27,28,29^ Our study does not speak directly to whether follow-up care improves outcomes, but it does suggest that rates of follow-up for discharged patients with mTBI may be lower than for other conditions. For example, 1 study found that 79% of Medicare beneficiaries with a wide range of conditions had a physician follow-up visit within 3 months of an acute care hospitalization, thereby reducing hospital readmission and decreasing health expenditures.^30^ In the case of mTBI, it is possible that its relatively low mortality rate and a general lack of care coordination after an ED visit contribute to low rates of follow-up even for patients experiencing symptoms.

### Limitations

Our study has several limitations. The sample size constrained the power of statistical analysis. The relatively small number of study sites and the fact that they were all university-affiliated, level I trauma centers may limit the generalizability of the results. We expect that hospital practice and procedures are a significant driver of follow-up care, and more work is needed to understand what can be done to improve rates of follow-up and availability of TBI-specific care. Another limitation is the potential for inherent biases associated with patient self-reporting of follow-up care and outcomes. In particular, patients who have experienced a brain injury may have impaired ability to recall receipt of educational materials or follow-up care. However, if patients do not recall receiving educational materials because of their head injury, this could mean that changes are needed in the process for delivering these materials. Our data also had relatively little information on polytrauma or other factors associated with injury that could have affected patient need for follow-up care. If patients had significant polytrauma not observed in the study, we expect that would increase the need for follow-up care, making our results even more noteworthy.

## Conclusions

We found low rates of follow-up care following mTBI with important variations across US level I trauma centers. The 2 study sites with facilities that specialized in TBI had the highest rates of follow-up care, and most patients who were followed up reported that it was helpful. Injury severity was associated with follow-up, although even patients experiencing persistent postconcussive symptoms and psychological distress often did not receive follow-up care. These results highlight the need for more rigorous and systematic follow-up for patients who experience TBI or concussion, including systems of care specifically designed to offer follow-up treatment to patients with mTBI.

## Notes

Supplement. **eTable.** Patient and Injury Characteristics: Full Sample and Subsample With Complete Follow-up Survey Responses at 2 Weeks and 3 Months
