# Supplementary material for: Assessment of Follow-up Care After Emergency Department Presentation for Mild Traumatic Brain Injury and Concussion: Results From the TRACK-TBI Study
Source: JAMA Netw Open. 2018 May 25;1(1):e180210. doi: 10.1001/jamanetworkopen.2018.0210 (PMC6324305; doi:10.1001/jamanetworkopen.2018.0210)
Supplement: Supplement. — eTable. Patient and Injury Characteristics: Full Sample and Subsample With Complete Follow-up Survey Responses at 2 Weeks and 3 Months [file jamanetwopen-1-e180210-s001.pdf]

Supplementary Online Content

Seabury SA, Gaudette É, Goldman DP, et al; TRACK-TBI Investigators. Assessment of follow-up care after emergency department presentation for mild traumatic brain injury and concussion: results from the TRACK-TBI study. *JAMA Netw Open.* 2018;1(1):e180210. doi:10.1001/jamanetworkopen.2018.0210

eTable. Patient and Injury Characteristics: Full Sample and Subsample With Complete Follow-up Survey Responses at 2 Weeks and 3 Months

This supplementary material has been provided by the authors to give readers additional information about their work.

© 2018 Seabury SA et al. *JAMA Network Open.*

In the eTable we report the summary statistics for the full set of subjects age ≥18 years with mTBI who were enrolled between February 26, 2014 and August 25, 2016 and compare them to the subsample who completed follow-up and answered the questions about educational materials and having seen a provider at 2 weeks and at 3 months, and finally the subsample that completed both sets of questions.

eTable. Patient and Injury Characteristics: Full Sample and Subsample with Complete Follow-Up Survey Responses at 2 weeks and 3 months

	**Full Sample**		**Subsample that Answered Follow-up Questions at 2 Weeks**		**Subsample that Answered Follow-up Questions at 3 months**		**Subsample Included in the study - Answered Follow-up Questions at 2 Weeks and 3 months**	
N	1316		1017		919		831	
**Patient Characteristics**								
Female	448	(34%)	350	(34%)	320	(35%)	289	(35%)
Non-Hispanic white	728	(55%)	570	(56%)	537	(58%)	483	(58%)
Average age (s.d.)	40.5	(17.2)	39.8	(16.9)	40.8	(17.1)	40.3	(16.9)
18-64	1171	(89%)	916	(90%)	814	(89%)	743	(89%)
65+	145	(11%)	101	(10%)	105	(11%)	88	(11%)
**Income/Insurance group**								
Less than $50K	693	(53%)	562	(55%)	504	(55%)	460	(55%)
$50K+	300	(23%)	256	(25%)	230	(25%)	212	(26%)
Unknown income	323	(25%)	199	(20%)	185	(20%)	159	(19%)
Uninsured (self-pay)	274	(21%)	208	(21%)	168	(18%)	153	(18%)
Insured	1042	(79%)	809	(80%)	751	(82%)	678	(82%)
Private insurance	672	(51%)	570	(56%)	507	(55%)	468	(56%)
Medicaid	133	(10%)	93	(9%)	103	(11%)	85	(10%)
Medicare	91	(7%)	69	(7%)	70	(8%)	61	(7%)
**Injury Severity**								
Lesion detected on CT scan	391	(30%)	296	(29%)	271	(30%)	236	(28%)
Average GCS at arrival (s.d.)	14.7	(0.5)	14.8	(0.5)	14.7	(0.5)	14.8	(0.5)
GCS=13	52	(4%)	36	(4%)	31	(3%)	25	(3%)
GCS=14	242	(18%)	180	(18%)	173	(19%)	148	(18%)
GCS=15	1022	(78%)	801	(79%)	715	(78%)	658	(79%)
**Mechanism of Injury**								
Road traffic incident	775	(59%)	609	(60%)	543	(59%)	494	(59%)
Incidental fall	330	(25%)	247	(24%)	232	(25%)	203	(24%)
Violence/assault	82	(6%)	67	(7%)	57	(6%)	52	(6%)
**Care Disposition Group**								
ED Discharge	404	(31%)	342	(34%)	298	(32%)	288	(35%)
Hospital admission - no ICU	564	(43%)	434	(43%)	397	(43%)	362	(44%)
Hospital admission - ICU	348	(26%)	241	(24%)	224	(24%)	181	(22%)

© 2018 Seabury SA et al. *JAMA Network Open.*
